# Identifying key conservation threats to Alpine birds through expert knowledge

**DOI:** 10.7717/peerj.1723

**Published:** 2016-02-29

**Authors:** Dan E. Chamberlain, Paolo Pedrini, Mattia Brambilla, Antonio Rolando, Marco Girardello

**Affiliations:** 1 Department of Life Sciences and Systems Biology, University of Turin, Turin, Italy; 2 Museo delle Scienze di Trento, Trento, Italy; 3 Sezione Zoologia dei Vertebrati, Museo delle Scienze di Trento, Trento, Italy; 4 Settore biodiversità e aree protette, Fondazione Lombardia per l’Ambiente, Seveso (MB), Italy; 5 Department of Bioscience, Aarhus University, Aarhus, Denmark

**Keywords:** Expert opinion, NMDS, Climate change, Grazing, Land abandonment, Risk assessment

## Abstract

Alpine biodiversity is subject to a range of increasing threats, but the scarcity of data for many taxa means that it is difficult to assess the level and likely future impact of a given threat. Expert opinion can be a useful tool to address knowledge gaps in the absence of adequate data. Experts with experience in Alpine ecology were approached to rank threat levels for 69 Alpine bird species over the next 50 years for the whole European Alps in relation to ten categories: land abandonment, climate change, renewable energy, fire, forestry practices, grazing practices, hunting, leisure, mining and urbanization. There was a high degree of concordance in ranking of perceived threats among experts for most threat categories. The major overall perceived threats to Alpine birds identified through expert knowledge were land abandonment, urbanization, leisure and forestry, although other perceived threats were ranked highly for particular species groups (renewable energy and hunting for raptors, hunting for gamebirds). For groups of species defined according to their breeding habitat, open habitat species and treeline species were perceived as the most threatened. A spatial risk assessment tool based on summed scores for the whole community showed threat levels were highest for bird communities of the northern and western Alps. Development of the approaches given in this paper, including addressing biases in the selection of experts and adopting a more detailed ranking procedure, could prove useful in the future in identifying future threats, and in carrying out risk assessments based on levels of threat to the whole bird community.

## Introduction

A large and increasing proportion of the world’s species are threatened with extinction, largely as a result of man’s activity (Butchart et al., 2010). Knowledge about the intrinsic and extrinsic factors that lead to population decline and ultimately species extinction can be used to help to direct management efforts and allocate limited resources to specific conservation interventions. It is necessary both to understand past and current threats that may have led to recent population declines or range contractions, but also to assess future risks for particular sets of species, their likely sensitivity to those risks, and ultimately to make provision for conservation action. This may be through designing and implementing management action, or simply designating potentially at-risk species for further research and monitoring.

Ideally, detailed autecological knowledge and demographic studies over a long-term would be used to assess likely future threats and extinction risks (Schurr et al., 2012); however, such a situation is very much an ideal, and there are relatively few species globally for which adequate data are available. Indeed, there are key knowledge gaps on the status and trends of a large proportion of the world’s species, whether considered threatened or not (Rands et al., 2010). This may be due to a lack of financial resources to collect such data, or the logistical difficulties of carrying out biodiversity research in challenging and inaccessible environments—and often these two aspects are not independent. Instead of relying on actual data, knowledge gaps in terms of potential threats to species have often been addressed through expert opinion (e.g., Martin et al., 2005; Sutherland, 2006), whereby the opinions of (usually several) experts in a given research field are elicited *in lieu* of, or in addition to, existing data. In the field of conservation biology, such approaches are typically designed in a way to facilitate data analyses (as here) which are subsequently used as the basis to guide decision making (e.g. O’Neill et al., 2008; Donlan et al., 2010; Czembor et al., 2011). Whilst there are a number of potential problems with such approaches, they can nonetheless provide useful information as long as they are analysed and interpreted correctly with suitable caveats (e.g., McBride, Fidler & Burgman, 2012; French, 2012; Sutherland & Burgman, 2015), and indeed they may be the only option for making ecological predictions in the absence of other information (Sutherland, 2006).

Globally, high altitude habitats have a relatively high biodiversity value, but in some areas at least are also under considerable threat from a range of factors, including changes in agricultural practices (e.g., Laiolo et al., 2004), increasing disturbance (e.g., Rolando et al., 2007) and climate change (Dirnböck, Essl & Babitsch, 2011). However, simply due to the logistical challenges of monitoring and research in mountain areas, there is a relative lack of data on distributions, population trends and even basic autoecology for species at high altitude. Birds are amongst the most well-studied taxa, and yet even in generally well-researched European countries, there is little information from the majority of species in high altitude habitats that could be useful in making predictions, e.g., detailed habitat requirements or factors determining variation in key demographic rates (Chamberlain et al., 2012). Furthermore, good long-term monitoring data for high altitude species is available in only a few countries (e.g., Maggini et al., 2011).

Much research into potential threats to populations of birds of the European Alps has focussed on effects of disturbance through winter sports (e.g., Arlettaz et al., 2007; Rolando et al., 2007) and on the effects of land abandonment (Laiolo et al., 2004), and more recently, climate change (Chamberlain et al., 2013; Braunisch et al., 2014; Maggini et al., 2014; Brambilla et al., 2015). Elevational range changes, likely resulting from climate change and/or land abandonment, have been described in a number of studies (e.g., Reif & Flousek, 2012; Pernollet, Korner-Nievergelt & Jenni, 2015), although, with some notable exceptions (e.g., Maggini et al., 2011), such studies have been largely based on restricted study locations. There is a need to identify a wider range of potential threats for the whole Alpine bird community across a larger area. We see expert opinion as an important step in gaining insight into the likely sensitivities of Alpine birds to future environmental change, and thereby to identify future conservation and research priorities. The specific aim of this study is to quantify qualitatively perceived conservation threats for European Alpine birds. We ask the following questions: (i) what are perceived by Alpine ornithologists as the most important threats across species? (ii) how are species distributed along gradients of perceived threat? (iii) Are there any spatial patterns of perceived threat at the community level?

## Materials & Methods

### Species and threat scores

Species regularly breeding in the European Alps were considered at altitudes above 1,700 m asl. We did not adopt an *a priori* definition of an ‘Alpine bird species.’ Rather, a complete species list was objectively derived from species recorded in two different data sets, both of which surveyed birds in a series of 10-minute point counts along altitudinal transects in the breeding season, from c. 1,700 m (the approximate altitude at which broadleaved forest does not occur) to c. 3,000 m, one in Piedmont and one in Trentino (full methods are given in Chamberlain et al., 2013). This included a total of 69 species (see Table S1) which we henceforth refer to as ‘Alpine birds.’ Species groups were defined based firstly on the predominant nesting habitat type used in the Alps at the altitudes considered, where ‘open’ indicates usually ground (or sometimes cliff)-nesting species occurring in Alpine grasslands, ‘treeline’ indicates species commonly occurring in mosaic habitats near to the treeline, ‘forest’ indicates species associated with mature forest, and ‘gen’ indicates generalist species occurring in a range of different habitats (often anthropogenic), and all other species which could not be classified on the basis of the former groups. Definitions were based on personal observations and experience, and also by the classifications used by the European Bird Census Council (http://www.ebcc.info). Species were also grouped according to taxonomy, namely Passeriformes (passerines), Accipitriformes (raptors), Galliformes, and other non-passerines (henceforth ‘non-passerines’), and according to conservation status based on SPEC category (BirdLife International, 2004). This classifies species on the basis of their conservation status in Europe, where SPEC1 indicates a species threatened in Europe and globally, SPEC2 indicates a species threatened in Europe, but not globally, and most of the global breeding population is within Europe, and SPEC 3 indicates a species which is threatened in Europe but not globally, and most of the global breeding population is outside of Europe.

To estimate perceived threats for our set of study species, we developed a questionnaire for evaluation by ornithologists with experience of working in the European Alps. Initial drafts of the questionnaire and instructions were sent to three experts in order to test the methodology and receive feedback. After this initial stage, the modified questionnaire was sent to 23 independent ornithologists (i.e., not including the authors) with experience of research and monitoring (i.e., on a wide range of species) in the Alpine region. The selection of experts was based on personal knowledge of ornithologists who had known experience of either scientific research or formal monitoring in the European Alps. The selection was therefore through personal contacts of the authors, rather than on objective criteria, the goal being to maximise the participation rate of the survey (see ‘Discussion’ for an assessment of advantages and disadvantages of this approach). A follow-up survey was carried out in order to collect further information on the level of experience of each expert, in terms of experience of research and/or monitoring Alpine birds, the number of years this experience covered, and whether they had carried out research into each of the ten categories.

**Table 1:** Hierarchical classification of threats to alpine birds.

1st level of threat	2nd level of threat^a^
1. Residential and commercial development	1.1. *Urbanization*—increases in housing, commercial and industrial areas
2. Agriculture/silviculture	2.1. *Forestry*—Changes to management of forests, e.g., harvesting strategies (clear-felling vs. selective logging), understorey clearance
	2.2. *Grazing*—Increases in sheep or cattle densities (e.g., changes to sward structure, disturbance to ground nesters)
3. Natural system modifications	3.1. *Abandonment*—Changes derived from pastoral abandonment (e.g., scrub encroachment, forest succession, changes to sward structure)
	3.2. *Climate Change*—Direct and indirect impacts of climate change
	3.3. *Fire*—Human induced fire
4. Biological resource use	4.1. *Hunting*—Both licensed and illegal hunting (includes persecution, e.g., of raptors) and fishing
5. Human intrusion and disturbance	5.1. *Leisure*—Direct disturbance and/or habitat modification due to winter sports (including piste creation and management and off-piste skiing/free riding), walking, biking, birdwatching, rock climbing, scrambling, paragliding.
7. Energy production and mining	7.1. *Mining*—Presence of open-cast mines or quarries
	7.2. * Energy*—Developments in renewable energy such as wind turbines, hydroelectric power (e.g., effects on water flow and quality, effects on riverside habitats), solar power.

**Notes.**

a Names of 2nd level threats given in italics are used as abbreviations for each threat in the text.

Respondents were asked to rank species according to a list of threats. A threat is defined as a factor that could have a negative impact on the population size or the distribution of a particular species at or above 1,700 m in altitude in the European Alps over a time frame of 50 years. We used a simplified version of the threats described in a unified scheme proposed by Salafsky et al. (2008), which comprises three hierarchical classes of threats which increase in specificity with each class. A full description of the threats is provided in Table 1. Respondents were only asked to rank threats at the more detailed level (2nd level). The ranking regimes were statements that define the negative impact that a potential threat can have on the population of a particular species (3 = severe negative effect, 2 = moderate negative effect, 1 = minor negative effect, 0 = no negative effect). This ranking is henceforth referred to as the threat score. In cases where a respondent deemed that there could be positive and negative effects under a given heading, he/she was asked to consider the net effect of each threat considered (e.g., if two factors under a given threat cancel each other out, then the threat score should be 0). Similarly, if there were two factors under a given threat that may have differing threat levels, the respondent was allowed to judge whether the effect of one over-rides the effect of the other, or whether an intermediate threat level was more appropriate. The questionnaire instructions are given in Supplemental Information 1.

The analytical objective was simply to allow a relative ranking of threats, rather than to try and make firm predictions of, for example, probability of regional or national extinction of a species. However, the interpretation of the level of threats, and in particular how it may vary across experts, is of interest in interpreting the results. Therefore, as part of the follow-up survey (see above), experts were also asked how they interpreted the different categories in relation to effects on species’ populations. The form for this survey is given in the Supplemental Information 2.

### Data analysis

Consistency in response between experts was assessed using Kendall’s coefficient of concordance (*W*). First, consistency in overall threat scores for the community was assessed by comparing the threat score across all species for each threat separately (i.e., calculation of *W* was based on a matrix of experts by species, for each threat). Similar calculations were also carried out to assess consistency of mean threat scores calculated for species groups according to taxonomy, habitat and conservation status (see below). The level of significance was set at *P* = 0.00125 (i.e., 0.05/40, to account for the large number of repeated statistical tests in Table 2). *W* was calculated using the kendall.global command in the R package vegan (Oksanen et al., 2015).

**Table 2:** Concordance in threat score between experts measured by Kendall’s concordance coefficient (*W*) for each threat category (see Table 1). Concordance was calculated for threat scores individual species (‘Species’), and for *T*_mean_ for species defined into groups based on taxonomy (‘Taxonomy’), main nesting habitat (‘Habitat’) and European threat status according to BirdLife’s categories of conservation concern (‘SPEC’). Coefficients are significantly different from 0 at *P* = 0.00125 (i.e., the Bonferroni adjusted significance level) unless given in parentheses. *N* = 19 for each coefficient.

Threat	Species	Taxonomy	Habitat	SPEC
Abandonment	0.53	0.70	0.87	0.86
Climate change	0.33	0.54	0.83	0.32
Energy	0.44	0.42	0.68	0.68
Fire	0.23	(0.04)	0.28	(0.19)
Forestry	0.43	(0.18)	0.66	0.42
Grazing	0.50	0.61	0.79	0.86
Hunting	0.46	0.77	0.75	0.64
Leisure	0.42	0.73	0.71	0.78
Mining	(0.15)	(0.08)	0.27	(0.13)
Urbanization	0.34	0.66	0.66	0.67

Data were analysed at the individual species level, and by defining species into groups defined according to taxonomy, habitat and conservation status (see below). For the species-level analysis, a consensus matrix across respondents was derived by calculating the mode of the scores for each species/threat combination, where significant concordance was indicated for a given threat. There were cases where there was no single mode value, hence two matrices were derived, one using minimum mode values and one using maximum values. Non-metric multidimensional scaling (NMDS) was initially used to summarise the major patterns of variation in the two matrices of threat scores in order to assess consistency between minimum and maximum mode. We decided to use NMDS over other methods, such as PCA, as it can handle non-Euclidean distance matrices (Legendre & Legendre, 2012). Because our input data consisted of categorical variables, the Gower general dissimilarity index (Gower, 1975) was used to construct a distance matrix. The basic objective of NMDS is to plot dissimilar objects that are far apart and similar objects close to one another in ordination space. NMDS makes no assumptions about normality or linearity of the underlying data. NMDS requires the user to specify a number of *m* dimensions to which the dataset is reduced. The *n* objects (here, species) are then placed and ranked in this pre-chosen space and an initial configuration of the objects in *m*-dimensional ordination space is computed. This configuration process is that used for an iterative arrangement. We used one-hundred random starts to find a stable solution to avoid local minima. Stress values, i.e., the sum of squared differences between fitted and original distances, were used to assess how well the configuration of points in reduced ordination space described the original distance matrix. Stress values, which can vary between 0 and 1, were used to rank species scores in terms of their goodness of fit.

We used Procrustes and PROTEST analyses (Peres-Neto & Jackson, 2001) to address the issue of variability within different forced consensus matrices by comparing the two NMDS ordinations performed on our data matrices based on the two different modes. Procrustes analysis works by scaling, rotating, and dilating one ordination solution and then superimposing it on a second ordination, maximizing the fit between corresponding observations of the two ordination configurations. The most frequently used method for Procrustean fitting is based on the least-squares criterion that minimizes the sum of the squared residuals (*m*^2^) between the two configurations; the *m*^2^ statistic is thus a measure of association (i.e., concordance) between the two configurations. This is the significance test for procrustes analysis to verify whether multivariate configurations match (Peres-Neto & Jackson, 2001). PROTEST extends Procrustes analysis by providing a permutation procedure to assess the statistical significance of the Procrustean fit (Peres-Neto & Jackson, 2001). PROTEST randomly permutes the original scores of the NMDS ordination so that scores can be assigned any of the values attributed to other species (Jackson, 1995). The *m*^2^ statistic is then recalculated for each permutation, and the proportion of the statistics smaller than or equal to the observed value provides the significance level of the test.

To summarise the general patterns in perceived threats to species groups (defined according to taxonomy, habitat or threat status), we calculated the mean species threat score *T*_mean_ for each group for each expert. The median *T*_mean_ was compared between groups using the Friedman test (i.e., analysing the expert × group matrix), or for comparisons with only two groups (relevant to SPEC status), the Wilcoxon test.

To assess the extent to which threat scores were applied by experts independently of published research on the ecology of each species in mountain environments, we correlated overall *T*_mean_ for each species, calculated across all threats and experts, with relevant citations for each species derived from a standardized search in Web of Science™. For each species individually, we searched for papers published since 1970 that contained both the species name and reference to the Alps or mountains, specifically ‘TS = (alps OR alpine OR mountain^∗^) AND TS = (“species English name” OR “species scientific name”)’. There are several cases where scientific names have changed over the past few decades (e.g., Willow Tit *Poecile montanus* was until recently *Parus montanus*). In such cases, searches were carried out including both old and new scientific names. Similarly, for species with alternative English names (such as Skylark *Alauda arvensis* or Sky Lark), all versions of a name were included in the search. We inspected each resulting reference list for non-relevant references (which were removed) and for any known references which were not included (in practice these were references that considered a range of species, rather than being species-specific studies, e.g., Laiolo et al., 2004; Caprio et al., 2011). The final total references were then correlated against *T*_mean_ for each species using Spearman rank correlation.

In order to explore spatial patterns of threat, we matched our threat scores to species distribution data for the Alpine region, derived from the European Atlas of Breeding Birds (Hagemeijer & Blair, 1997). Although these distributional data have a rather coarse resolution (50 × 50 km), they are the best available data covering the whole Alpine region. An overall threat score was calculated for each 50 × 50 km square by taking the mean of the overall threat scores of all the species present in that square. We tested for the existence of any latitudinal or longitudinal gradients of threats using Spearman’s rank correlation coefficients.

## Results

We received 20 completed questionnaires from researchers working in Italy (*n* = 15), Austria (*n* = 3), Switzerland (*n* = 2) and Spain (*n* = 1). One questionnaire was omitted from the analysis due to errors (the expert did not follow the recommended scoring system), leaving a sample of 19. The experts were from a range of professions, with seven from universities, five from research institutes (including museums), four freelance professionals and two working for national or regional parks. One expert specified joint employment for a university and a national government. The sample of experts was strongly male-biased with only a single female representative. For the follow-up questionnaire, there were twelve responses (i.e., 60% of the original respondents). Of this sub-sample, the average age was 48.5 years (range 36–63), and the average years of experience on Alpine research or monitoring was 20 years (range 6–45). Of this subsample, eight reported experience of research on *Hunting*, seven each on *Forestry* and *Climate Change*, five on *Abandonment*, four each on *Grazing*, *Leisure* and *Urbanization*, three on *Energy*, one on fire and none on mining. In terms of interpretation of threat levels, the experts were generally highly consistent in defining severe threats as leading to local extinction and reductions in distributions (10 out of 12). The moderate category had greater variation in definition, but for the most part was interpreted in terms of strong population declines (*n* = 9), but also of changes in distribution (*n* = 2), or simply the geographical extent over which the decline occurred (*n* = 1). The minor category was generally defined as being detectable or statistically significant, but without leading to consequences for the population (*n* = 10). (See Supplemental Information 2 for full responses.)

**Figure 1:**
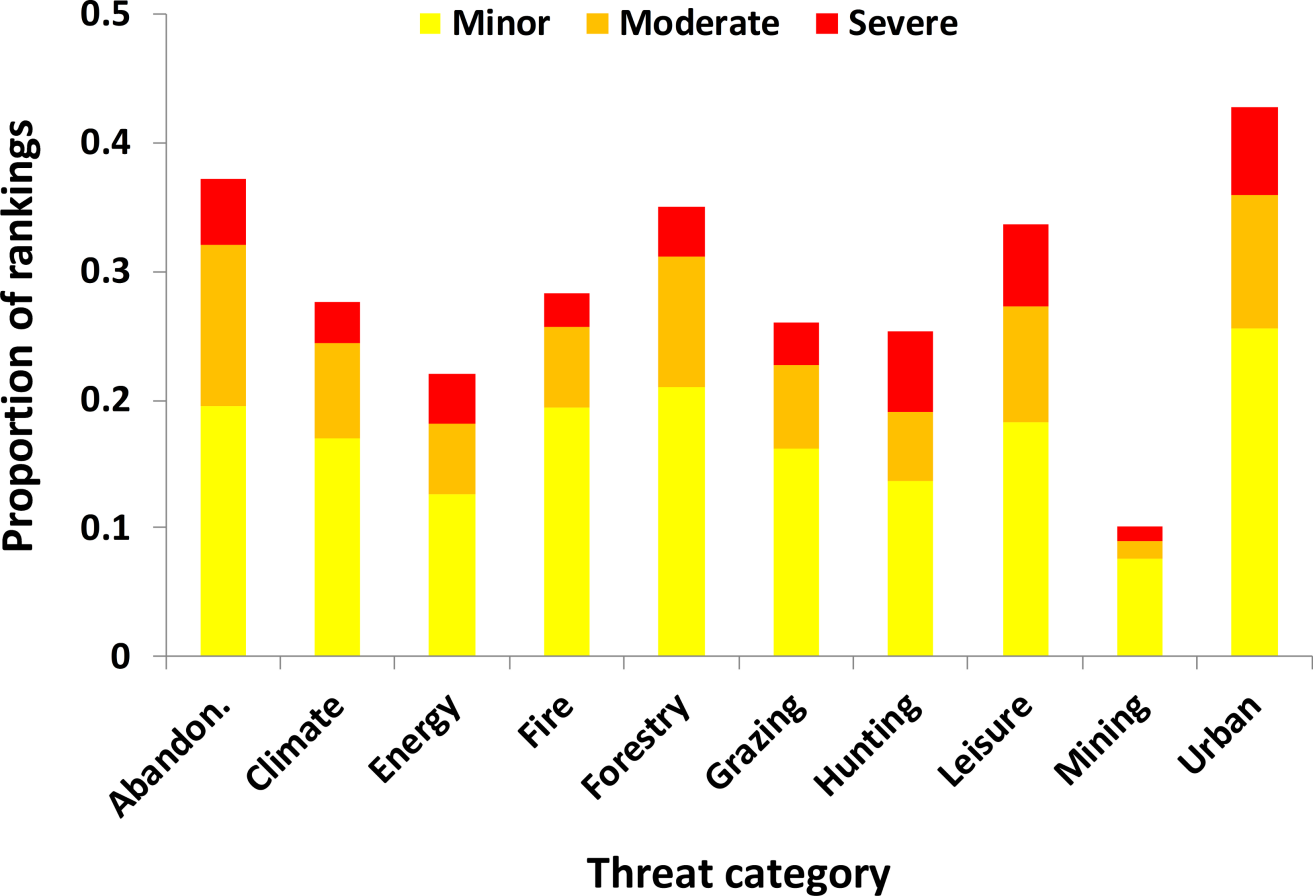
The proportion of species ranked as under minor (yellow), moderate (orange) or severe (red) threat in the future under different threat categories across 19 experts. Each column is based on 1,311 rankings (19 experts, 69 species). Further details of threat categories are given in Table 1.

A summary of the extent of perceived threat for each category was made by calculating the percentage of species that were ranked as at risk of minor, moderate or severe threat across all 19 experts for each threat category separately (Fig. 1). Considering non-zero threats (i.e., disregarding whether minor, moderate or severe), *Urbanization* was perceived as the most common threat across experts (c. 43% of responses were perceived as at least a minor threat across all experts), but *Abandonment*, *Forestry* and *Leisure* were also commonly perceived as threats (>30%). Other threat categories (*Fire*, *Climate Change*, *Grazing*, *Hunting* and *Energy*) were perceived as threats fairly commonly (22–28% of responses). *Mining* was notably lower than the other threat categories (19%). The patterns were slightly different when considering only the higher threats (moderate or severe). *Abandonment*, *Urbanization*, *Leisure* and to a lesser extent *Forestry* were perceived as relatively moderate or severe. However, there were some threat categories that, whilst having a high percentage of non-zero threat scores, were less commonly considered moderate or severe, in particular *Grazing*, *Energy* and *Fire* had fewer than 10% of responses classified as moderate or severe.

### Concordance

*W* for each individual threat, over all species and according to species groups, is shown in Table 2. There was a good level of concordance among experts for most threats for all of the different classifications, and most values of *W* were significantly different from zero. Concordance for all individual species was generally lower than for species groups, notably for *Mining* (which was not significantly different from zero), *Fire*, *Climate Change* and *Urbanization* (*W* < 0.40). However, some caution is needed in interpreting the latter results, given that there were many species, but only four possible threat scores, meaning that a large number of tied ranks were inevitable in the analysis. Nevertheless, we include these results for completeness. *Mining* and *Fire* (and to a lesser extent *Forestry*) had consistently low concordance overall, which was often not significantly different from zero. Given the significant and often high levels of concordance among experts, in the following analyses we use statistics summarised across experts to analyse general trends in threat score, except for *Mining* and *Fire*, which were given a threat score for very few species by only a few experts, and this allocation was not consistent across experts (Table 2), therefore these are not considered further.

### Species-level analysis

The NMDS ordinations lead to satisfactory projections into two-dimensional space indicated by relatively low stress values (maximum mode values = 0.061, minimum mode values = 0.065). A comparison of the two NMDS ordinations (i.e., maximum or minimum mode) by means of a PROTEST analysis revealed that these were virtually identical (*m*^2^ = 0.94 *P* < 0.001), so we only report the results from the NMDS performed on the consensus matrix containing the maximum values of the mode.

**Figure 2:**
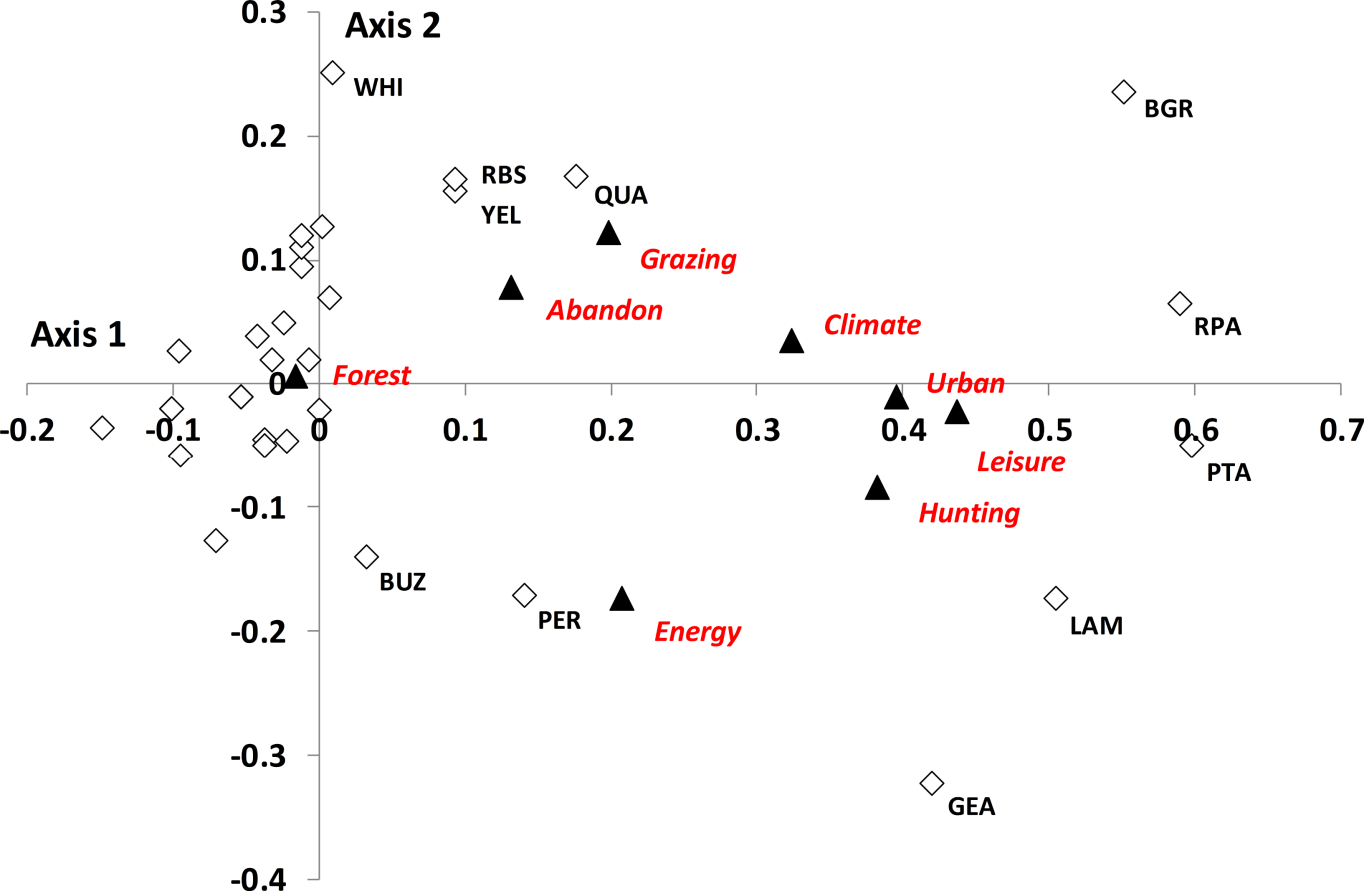
Results from the NMDS analysis of the species by threat matrix. Filled triangles are scores for each threat on the two NMDS axes. Open diamonds are scores for each species distributed along a threat gradient, codes identify species. For clarity, species with low scores on both axes (i.e., those clustered around the origin) are not identified to species. Species codes are: BGR, Black Grouse; BUZ, Buzzard; GEA, Golden Eagle; LAM, Lammergeier; PER, Peregrine Falcon; PTA, Ptarmigan; QUA, Quail; RBS, Red- Backed Shrike; RPA, Red-legged Partridge; WHI, Whinchat; YEL, Yellowhammer.

A bi-plot of the threat scores on the first two axes, and the species categories plotted in the same dimension, is shown in Fig. 2 (the modes upon which this figure is based are given in Table S2). Axis 1 was associated with *Urban*, *Leisure* and *Hunting*. Axis 2 was associated with *Abandonment* and *Grazing* and negatively with *Energy*. The majority of species were characterized by low threat scores and hence were clustered around the origin, with no link to any particular threat (these are not shown on Fig. 2). Species associated with axis 1 were those with the highest perceived threats for *Urbanization*, *Leisure* and *Hunting*, and to a lesser extent *Climate Change* (i.e., those to the right of the figure), and included raptors (Golden Eagle *Aquila chrysaetos* and Lammergeier *Gypaetus barbatus*) and Galliformes (Black Grouse *Tetrao tetrix*, Ptarmigan *Lagopus muta*, Rock Partridge *Alectoris graeca*). In a similar manner, the major trend along axis 2 was a division of raptors (Golden Eagle, Lammergeier, Buzzard *Buteo buteo*, Peregrine Falcon *Falco peregrinus* in the lower half of the graph), suggesting *Energy* as a major perceived threat, and species of open or transitional habitat (e.g., Black Grouse, Quail *Coturnix coturnix*, Whinchat *Saxicola rubetra*, Red-backed Shrike *Lanius collurio*, Yellowhammer *Emberiza citronella*, in the upper half of the figure) suggesting *Abandonment* and *Grazing* as the greatest perceived threats.

**Figure 3:**
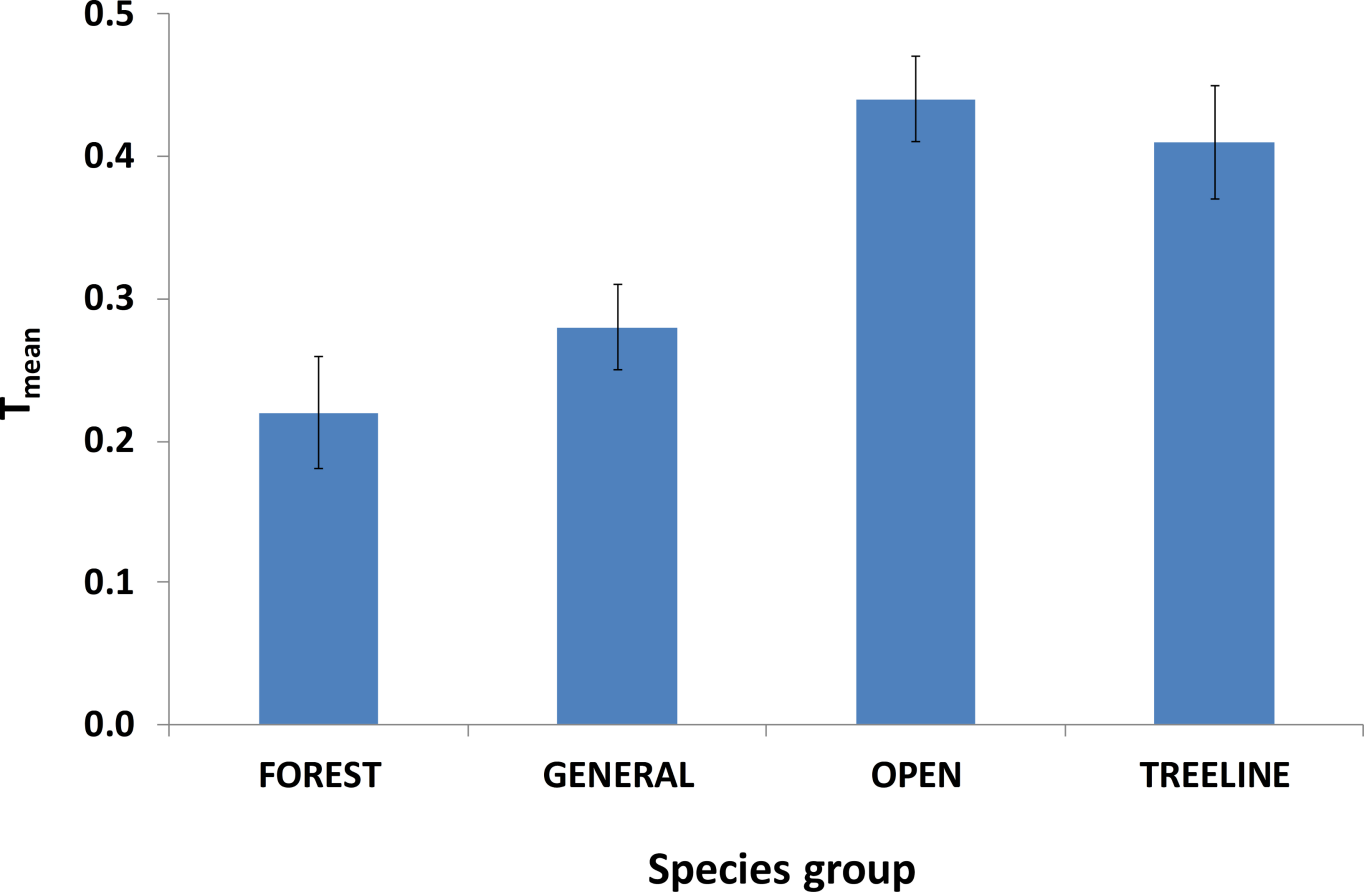
Mean (±se) *T*_mean_ for species groups defined according to nesting habitat. See Table S1 for individual species in each group.

### Species group analysis

**Figure 4:**
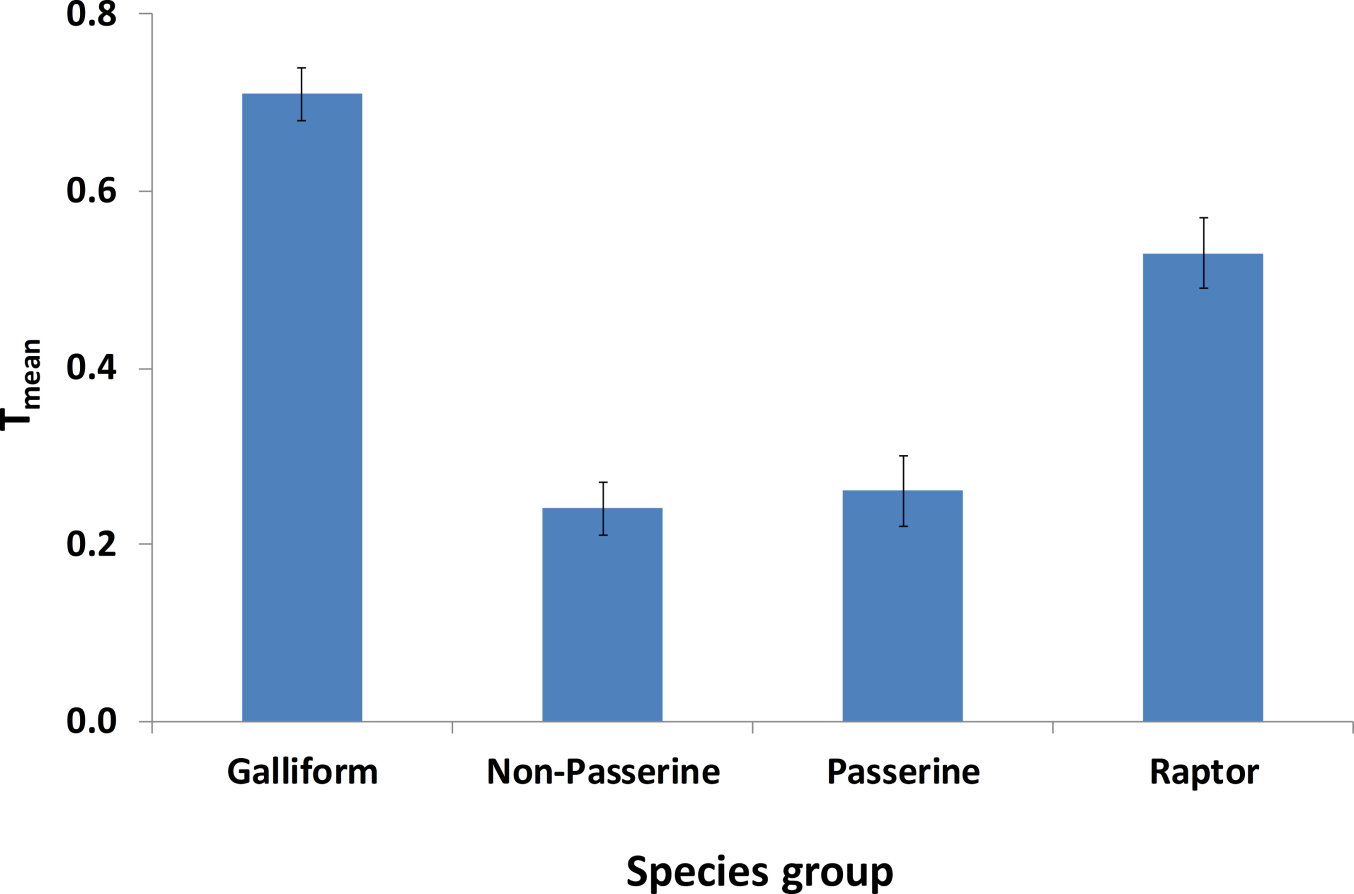
Mean (±se) *T*_mean_ according to taxonomic group. See Table S1 for individual species in each group.

Average *T*_mean_ for species groups defined according to habitat calculated across experts is shown in Fig. 3. Open habitat and treeline habitat species groups had on average the highest *T*_mean_. The difference between groups was highly significant (Friedman test, ${\chi }_{3}^{2}=48.1$, *p* < 0.001). When defined according to taxonomic group, *T*_mean_ for Galliformes and raptors were far higher than those for passerines and non-passerines (Fig. 4), and the difference was highly significant (Friedman test, ${\chi }_{3}^{2}=51.4$, *p* < 0.0001). The effect of habitat was in part influenced by the strong effects on raptors and Galliformes. When species from these two groups were removed from the habitat comparison, there was a notable decrease in *T*_mean_ for open habitats (with raptors and Galliformes =0.44 ± 0.03 sd, *n* = 19; without =0.32 ± 0.04, *n* = 19) and to a lesser extent for generalist species (with =0.28 ± 0.03, *n* = 19; without =0.23 ± 0.03, *n* = 19) and for treeline habitat species (with =0.41 ± 0.04, *n* = 19; without =0.37 ± 0.04, *n* = 19), the latter being the habitat with the highest *T*_mean_ without raptors and galliformes. There was no change in *T*_mean_ for forest species. The difference between groups was still significant without raptors and Galliformes (Kruskal–Wallis test, ${\chi }_{3}^{2}=42.9$, *p* < 0.001).

Of the 69 species considered, 5 were classed as SPEC2 and 12 were classed as SPEC3 (Table S1). *T*_mean_ for species classified according to SPEC status across experts was higher for those listed as of conservation concern (i.e., combining SPEC2 and SPEC3 species; mean ±se = 0.45 ± 0.03, *n* = 19) than unlisted species (mean ±se = 0.27 ± 0.03, *n* = 19). The difference was highly significant (Wilcoxon test, *W* = 173, *p* = 0.001). There was no significant correlation between the number of relevant citations and *T*_mean_ (*r*_*s*_ = 0.21, *n* = 69, *p* = 0.08).

**Figure 5:**
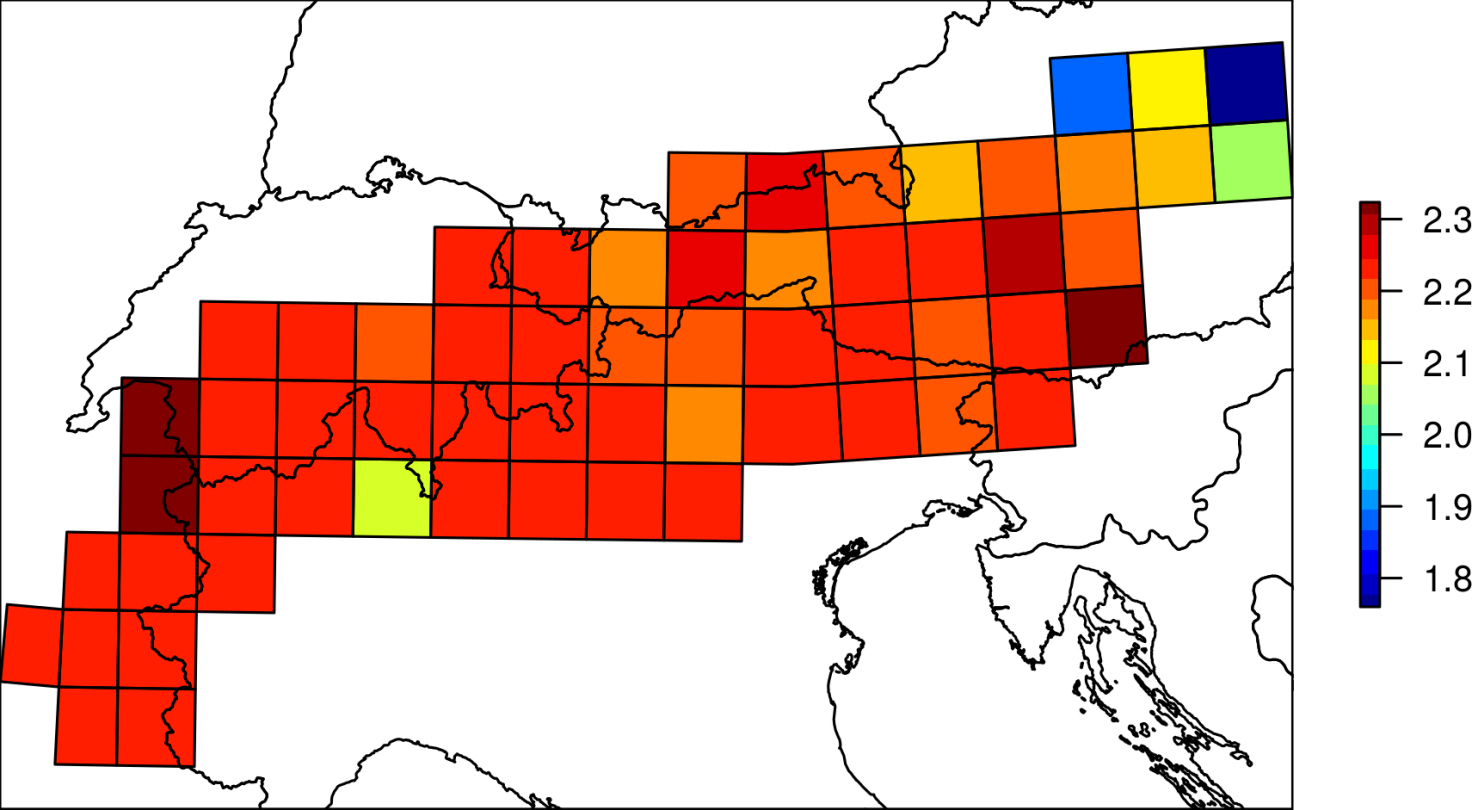
Spatial variation of threat scores at the community-level. Values represent mean threat scores for the species present in each 50 ×50 km square, colour-coded according to the scale bar on the right.

The mapping exercise highlighted a moderate amount of spatial variation in the threat scores at the community-level (Fig. 5). Both longitudinal and latitudinal gradients of threat were statistically significant (rho_lat_ = − 0.50 *P* < 0.001, rho_lon_ = − 0.49*p* < 0.001). Average threat scores tended to decrease when moving eastwards and southwards.

## Discussion

The greatest perceived threats to populations of Alpine birds over the next fifty years were mostly associated with land management, in particular urbanization, land abandonment, changes in forestry practices and changes in leisure activities. The species groups considered most threatened were clearly Galliformes and raptors. There was a high degree of concordance between experts at the species group level, and a reasonable level of concordance at the species level, indicating that responses were generally consistent across experts.

### Caveats on interpretation

Caution needs to be taken when interpreting expert knowledge surveys (Martin et al., 2012). A particular caveat for our study was that some threats may not be independent. For example, climate change may result in less snow in winter, which in turn may mean that ski pistes are constructed at higher altitudes, increasing disturbance in these areas (Elsasser & Messerli, 2001). In this case, although the ultimate cause may be climate change, an expert may rank leisure as a greater threat. We were aware of this, but decided not to provide guidance to the experts to avoid influencing their scores. However, in such cases we feel that the experts identified the main proximate threats.

A second caveat is that sample size was not large (19 experts completed the questionnaire correctly), although it was comparable to many other (e.g., *n* = 20, Martin et al., 2005; *n* = 10, O’Neill et al., 2008; *n* = 8, Czembor et al., 2011; *n* = 16, Lukey et al., 2011), but by no means all (e.g., *n* = 244, Donlan et al., 2010), published expert knowledge surveys. Rather than the small sample size *per se*, the geographical distribution of experts and small sample sizes for certain regions may present more of a bias. Despite that we requested that experts judge threats for the whole Alpine region, and not just their own country or region, we cannot rule out that opinions were influenced by regional issues. Geographical variation may cause different experts to assign different threats depending on local, regional or national land use trends. For example, land abandonment is a major issue in the Western Italian Alps (e.g., Motta, Morales & Nola, 2006), whereas intensification of grassland management is occurring in some parts of Switzerland, at least towards the lower altitudinal limit considered here (e.g., Andrey et al., 2014).

There was no evidence overall that the experts’ ranking was associated with the extent of knowledge on the species, as measured by the number of citations. Furthermore, for the experts that responded to the follow-up survey, there was no correlation between the number of cases (i.e., all species across experts, as in Fig. 1) classed as a non-zero threat and the number of experts claiming research experience for a given threat (Spearman correlation *r*_*s*_ = 0.32, *n* = 12, *p* = 0.40), nor was there any correlation when considering only species classed as moderate or severely threatened (*r*_*s*_ = 0.52, *n* = 12, *p* = 0.12). However, it should be noted that the two threats with the lowest concordance, *Fire* and *Mining*, were also those for which the experts had hardly any experience. We cannot therefore completely discount that for some species, the experts were influenced by their own research experience, or by the published literature in assigning threats. It should also be pointed out, however, that a number of species were perceived as more threatened than average for which little research had been carried out in alpine environments (e.g., Quail, Wheatear *Oenanthe oenanthe*, Whinchat, Red-backed Shrike, Yellowhammer all <10 citations and *T*_mean_ ≥ 0.57, the overall mean across species being 0.44). These species therefore may warrant further research in the future.

The respondents were selected based on the choice of the authors, often through previous professional collaborations, rather than being selected in any systematic way. This may have biased the outcomes. For example, most experts were Italian, male and middle-aged. However, there is likely to be a trade-off between the response rate and the degree to which experts are selected based on wholly objective criteria (as per Czembor et al., 2011). Indeed, the response rate here was exceptionally high (83%, not including the incorrectly compiled questionnaire) compared to other similar studies (e.g., 17%, Czembor et al., 2011; 32%, Lukey et al., 2011). Even in these studies, it seems plausible to expect that those experts that responded would have been dominated by those known to the authors. Nevertheless, a more diverse panel in terms of nationality, age and gender would have been desirable, in keeping with the recommendations of Sutherland & Burgman (2015). The extent to which such biases can affect the outcomes of expert knowledge surveys would need a large sample of both individually selected and objectively selected experts.

### Key perceived threats

Several threats that were perceived as most important were associated with land use changes in the Alps that have been ongoing for several decades. Land abandonment by human populations has been an ongoing process in many parts of the Alps over the course of the past century (Cernusca, Tappeiner & Bayfield, 1999), and this has been accompanied by a decrease in farming activity resulting in encroachment of shrubs and subsequently forest in formerly open areas. Whilst this may mean a return to a more ‘natural’ state, there is nevertheless evidence that the loss of the cultural landscape mosaic of shrubs, trees and grassland in the Alps has had a negative overall impact on biodiversity (e.g., Laiolo et al., 2004). At the same time, many areas have moved away from farming to leisure activities, in particular winter sports, which have several reported negative effects on biodiversity (e.g., Arlettaz et al., 2007; Rolando et al., 2007). Whilst the urban working population of Alpine settlements declined over the course of the last century (Cernusca, Tappeiner & Bayfield, 1999), urbanization was perceived as a continuing threat largely due to developments to accommodate the current main economic contributor, tourism.

Climate change is undoubtedly considered one of the major potential threats to biodiversity globally (Parmesan, 2006), and also specifically to mountain biodiversity (Sekercioglu et al., 2008). However, climate change was rarely ranked as moderate or severe, and had relatively low concordance for individual species and for species groups defined according to SPEC status, despite that recent models have indicated potential negative effects of climate change on alpine birds in the future (Chamberlain et al., 2013; Braunisch et al., 2014; Maggini et al., 2014; Brambilla et al., 2015). This may reflect the fact that most models of climate change impacts on biodiversity are considered over a relatively longer term (Chapman et al., 2014) and are therefore perceived as being less relevant to current conservation problems. The results concur with the view that habitat degradation and loss are the key general threats to terrestrial biodiversity (e.g., Sala et al., 2000; Jetz, Wilcove & Dobson, 2007).

### Species perceived as threatened

Galliformes and raptors were the two groups perceived to be most threatened, both in terms of individual (NMDS) and group-level (Fig. 4) analyses. Both of these groups are quite sensitive to disturbance (e.g., Arlettaz et al., 2007; Watson, 2010) and are also often killed either for, respectively, food (grouse, Watson & Moss, 2008) or illegal shooting (raptors, Pedrini & Sergio, 2001). Historically, it seems likely that raptor populations in the Alps have been limited by persecution, although this threat has declined in recent years (e.g., Fasce et al., 2011). Furthermore, large raptors are likely to be relatively vulnerable to collisions with wind turbines (e.g., Madders & Whitfield, 2006; Bellebaum et al., 2013), hence changes in energy generation are considered a threat. Neither *Hunting* nor *Energy* were perceived as the commonest overall threats, but they clearly were perceived as being very important for particular species groups.

Other species groups (passerines and other non-passerines) had on average much lower threat scores, with many not being considered as threatened by many of the factors considered. In terms of habitat groups, this was also true for generalists (often widespread adaptable species found in small numbers at the limit of their altitudinal range in our study) and forest species. Open habitat species were perceived as the most threatened habitat group overall, although this was largely driven by a relatively small number of raptors and Galliformes. When these were removed, the remaining species (including high altitude specialists such as Alpine Chough *Pyrrhocorax graculus* and Alpine Accentor *Prunella collaris*) were considered not highly threatened. There were higher scores for species associated with mosaic habitats close to the treeline, such as Red-backed Shrike, Whinchat and Yellowhammer, reflecting perceived threats of land abandonment and grazing intensity (e.g., Brambilla, Rubolini & Guidali, 2007).

The experts were requested to rank each species on the basis of likely future impacts using a simple 4-level ranking. Whilst other similar surveys have used far more detailed rankings (e.g., up to 10 levels—Donlan et al., 2010), we adopted a deliberately simple approach in the hope of maximising participation. Whilst the experts showed good levels of consistency in their interpretation of the different ranks, there remain two potential drawbacks with the approach. First, there was no scope for including an unknown category in the scoring system. We cannot therefore discount that a zero rank may have represented an unknown effect rather than no effect, but we still feel that the method identifies the species that are currently considered as priorities.

Second, the approach adopted focussed on perceived negative effects. However, some of the drivers identified may also have positive effects for some species. Both climate change and land abandonment may have positive effects for forest species due to forest expansion (e.g., Chamberlain et al., 2013). Here, for the most part, forest species had low threat scores, so positive effects in these species do not affect the conclusions concerning the majority of threatened species identified (although it should be noted the *Forestry* had relatively low concordance). Similarly, urbanization may benefit some generalist species, but again, these were species with low or zero threat scores and typically generalists more usually associated with lower altitudes. Increased hunting pressure, whilst disproportionately affecting grouse and raptors, may nevertheless have positive effects on some, particularly prey, species. In general, whilst we acknowledge that some positive effects may cancel out or exceed negative effects in some species, such effects are likely to be more relevant to generalist and/or lower altitude species (i.e., forest species)—the key species groups identified as being under threat are unlikely to show any compensatory positive effects from any of the threats considered.

In general, the perception of the level of threat identified in the survey was in accord with the conservation status of the species considered, in that species classified as of conservation concern by BirdLife International had higher *T*_mean_ (see Table S1 for SPEC classifications of the species considered). Therefore in general, the experts were identifying those species of most current concern as having higher levels of perceived threat. There may of course be some effect of prior knowledge of the experts, i.e., they may have been more likely to consider a species as threatened if they knew it was already classified as of conservation interest. Nevertheless, there were also 25 species with no SPEC listing that were identified as facing some threat (i.e., that appeared in Table 2). Of particular interest are those species that are not currently classified as SPECs, but which had relatively high threat scores (e.g., Buzzard, Peregrine Falcon, Ptarmigan, Whinchat and Yellowhammer). Whilst some of these species may not be of conservation concern at present, they may be species that should be considered as higher priorities for research and conservation actions before problems arise in the future.

### Geographic distribution of perceived threat

By combining threat scores for individual species with their distribution across the Alps, we were able to produce a map of geographical variation in overall threats to the bird community. This suggested that Alpine bird communities had more threatened species in the north and west of the area. Such an approach could be used as a basis for identifying areas that have bird communities that may be sensitive to the threats identified, and therefore may be candidate locations for species protection through active conservation management or restrictions on activities (leisure, agriculture, energy generation) that may threaten bird populations in those areas. The scale at which the threat map was produced was limited by the availability of the underlying bird distribution data, and therefore may be of use in guiding regional conservation strategies at a relatively large scale in that it identifies regions that are worthy of more detailed consideration (e.g., suitable locations for the creation of protected areas). However, we feel that the approach has great potential in developing risk assessments for environmental change which take a community-level approach, rather than focussing on individual species of conservation concern, and that it could be developed further given the availability of finer-scale data.

## Conclusions

The major threats to Alpine birds identified through expert knowledge were those that, for the most part, could be addressed with concrete actions such as targeted grazing or shrub clearance to maintain open habitats, adopting more sympathetic forestry practices (e.g., Braunisch et al., 2014), restricting, or planning more environmentally sustainable, urban developments, and better management of potential disturbance factors (e.g., winter sports and other leisure activities) at high altitudes. There was less emphasis on threats that have a higher degree of uncertainty, at least in part due to lack of knowledge (e.g., mining and fire), and therefore for which it is difficult to formulate a management strategy. Despite that most climate change research is based on long-term forecasts and concerns direct effects (Chapman et al., 2014), climate change was perceived as a minor threat. Even when asked for long-term predictions (50 years in this case), threats associated with current conservation issues seemed to be perceived as more important. Policy decisions are typically taken on relatively short timescales. Longer-term threats may indeed be of lesser importance, but it may also be that, despite a huge body of research on potential consequences of climate change for biodiversity, scientists and practitioners still do not fully consider long-term issues.

Whilst there are caveats on the interpretation of expert knowledge surveys, including some potential biases present in this survey, we believe that this approach has highlighted some conservation priorities in the Alps, and has also identified some species that may be of concern in the future, despite not yet being of conservation interest. Development of the approaches given in this paper, including addressing biases in the selection of experts and adopting a more detailed ranking procedure, could prove useful in the future in identifying future threats, and in carrying out risk assessments based on levels of threat to the whole bird community.

## Supplemental Information

10.7717/peerj.1723/supp-1 Supplemental Information 1 Expert responses to questionnaire survey

10.7717/peerj.1723/supp-2 Supplemental Information 2 Questionnaire instructions

10.7717/peerj.1723/supp-3 Supplemental Information 3 Follow-up Survey Follow-up survey form and experts’ definitions of threat levels.

10.7717/peerj.1723/supp-4 Table S1 A list of all species considered in the questionnaire, and the ecological group assigned to each species *T*_mean_ is the arithmetic mean of the threat score across the 19 experts who completed the survey correctly. Groups were defined according to (i) taxonomic order, which comprised Galliformes, Raptors (Accipitriformes), other non-passerines (Piciformes, Apodiformes and Cuculiformes) and Passerines; (ii) the predominant nesting habitat type used by the species in the Alps at the altitudes considered, comprising Forest (species associated with mature forest), General (generalist species occurring in a range of different habitats, often anthropogenic, and all other species which could not be classified on the basis of the former groups), Open (usually ground (or sometimes cliff)-nesting species occurring in Alpine grasslands) and Treeline (species commonly occurring in mosaic habitats around the treeline). Definitions were based on personal observations and experience, and also by the classifications used by the European Bird Census Council (  http://www.ebcc.info). SPEC is the conservation status of each species in Europe according to BirdLife’s SPEC classification (BirdLife International, 2004), where 0 indicates the species is not listed as threatened, 2 indicates a species threatened in Europe, but not globally, and most of the global breeding population is within Europe, and 3 indicates a species which is threatened in Europe but not globally, and most of the global breeding population is outside of Europe (there were no SPEC1 species). Cites is the number of published articles within a mountain environment since 1970 for a given species following a standardised search in Web of Science™ . All species were recorded at least once in point counts surveys carried out in Piedmont in 2010 to 2012 (*n* = 271 points) and Trentino (*n* = 208 points) in 2011.

10.7717/peerj.1723/supp-5 Table S2 Consensus threat scores used in the NMDS analysis The analysis included the 39 bird species with at least one non-zero mode of threat score.

## Additional Information and Declarations

### Data Availability

The following information was supplied regarding data availability: The questionnaires, ranking the threats for each of the 19 experts, are provided in the Supplemental Information as a single .csv file where each expert is identified with a letter. These are listed in random order so as to maintain anonymity.
