# Supplementary material for: Identifying key conservation threats to Alpine birds through expert knowledge
Source: PeerJ. 2016 Feb 29;4:e1723. doi: 10.7717/peerj.1723 (PMC4782807; doi:10.7717/peerj.1723)
Supplement: Supplemental Information 2 [file peerj-04-1723-s002.docx]

**Supplemental Information 1. Questionnaire instructions.**

| **INSTRUCTIONS FOR COMPILING THE QUESTIONNAIRE** |
| --- |
| *Definition of threat:*  A threat is defined as any of the factors listed below that could have a **negative** impact on the population size or the distribution of a particular species **at or above 1700m** in altitude in the European Alps over a fairly short time frame (within 50 years). The responses should consider the Alps as a whole (i.e. not country-specific). |
| *Description of threats:*  T1-Pastoral abandonment  Changes derived from pasture abandonment (e.g. scrub encroachment, forest succession, changes to sward structure)  T2-Overgrazing  Increases in sheep or cattle densities (e.g. changes to sward structure, disturbance to ground nesters)  T3-Forest management  Harvest strategy (e.g. selective vs block logging), understorey management, infrastructure (e.g. forestry tracks)  T4-Urbanisation  Habitat loss or modification due to spread of urban areas, transport infrastructure, winter sports infrastructure (hotels, ski resorts etc), power lines  T5-Leisure activities  Direct disturbance and/or habitat modification due to winter sports (including piste creation and management and off-piste skiing/free riding), walking, biking, birdwatching, rock climbing, scrambling, paragliding.  T6-Hunting  Includes persecution (e.g. of raptors) and fishing  T7-Renewable energy  Wind turbines, hydroelectric power (e.g. effects on water flow and quality, effects on riverside habitats), solar power  T8-Mining  Presence of open-cast mines or quarries  T9-Fire  Human-induced fires  T10-Climate change  Direct and indirect impacts of climate change |
| *Species selection:*  Species were selected objectively based on all species recorded on transect surveys carried out in Trentino and Piedmont in 2010 and 2011. The surveys recorded almost all species that occur in the breeding season at relatively high altitudes (i.e. above 1700m) in these areas. |
| *Scoring regime:*  The scoring regimes are statements that define the **negative** impact that a potential threat can have on the population of a particular species. In cases where it is deemed that there could be positive and negative effects under a given heading, we ask that the **net effect** of each threat is considered (e.g. if two factors under a given threat cancel each other out, then the score should be 0). Similarly, if there are two factors under a given threat that may have differing threat levels, we leave it up to the respondent whether the effect of one over-rides the effect of the other, or whether an intermediate threat level is more appropriate.  3 *:* very big negative effect  2 : big negative effect  1 : small negative effect  0 : no negative effect |
