# Supplementary material for: Identifying key conservation threats to Alpine birds through expert knowledge
Source: PeerJ. 2016 Feb 29;4:e1723. doi: 10.7717/peerj.1723 (PMC4782807; doi:10.7717/peerj.1723)
Supplement: Supplemental Information 3 — Follow-up survey form and experts’ definitions of threat levels. [file peerj-04-1723-s003.docx]

**Supplemental Information 2. Follow-up survey form and experts’ definitions of threat levels**

**NAME**:

| **COUNTRY** |  |
| --- | --- |
| **UNIVERSITY/INSTITUTE/COMPANY NAME** |  |
| **YEARS EXPERIENCE IN RESEARCH** |  |
| **YEARS EXPERIENCE IN ALPINE ECOLOGY** |  |
| **CARRIED OUT SURVEY WORK IN MOUNTAINS (YES/NO)** |  |
| **AGE** |  |
| **SEX** |  |
| **RESEARCH CARRIED OUT ON: (YES/NO)** |  |
| **LAND ABANDONMENT** |  |
| **CLIMATE CHANGE** |  |
| **RENEWABLE ENERGY** |  |
| **FIRE** |  |
| **FORESTRY** |  |
| **GRAZING** |  |
| **HUNTING** |  |
| **LEISURE ACTIVITIES** |  |
| **MINING** |  |
| **URBANIZATION** |  |
| **DO YOU WISH TO REMAIN ANONYMOUS?**  **(I.E. IN THE ACKNOWLEDGEMENTS)** |  |

**HOW DID YOU INTERPRET THE THREAT LEVELS OF ‘SEVERE’, ‘MODERATE’ AND ‘MINOR’ NEGATIVE EFFECT ON THE POPULATION?**

**Responses to Follow-Up Survey on definitions of severe, moderate and minor impacts on populations of Alpine Birds**

Reponses given in to the question: *HOW DID YOU INTERPRET THE THREAT LEVELS OF ‘SEVERE’, ‘MODERATE’ AND ‘MINOR’ NEGATIVE EFFECT ON THE POPULATION?*

**Expert A**

Severe: pushing a population to the brink of local extinction (example: black grouse in some major tourist resorts in the Swiss Alps)

Moderate: causing a serious decline of a local or regional population

Minor: shallowly impacting the demography of a population but without representing a real threat for its survival

**Expert B**

SEVERE: Significant and persistent decline over long period (i.e. 10 or more years), leading to species range shrinkage and/or elevation shift

MODERATE: Significant and persistent decline of species density over long period (i.e. 10 or more years), without range contraction and elevation shift

MINOR: Local population decline occurring seasonally or transiently

**Expert C**

Severe: Negative effect bringing to total extinction at specified scale and/or to a change of the status at national or macro-regional level.

Moderate: strong and evident decline (approximately 30-70%) but not to the extinction at specified scale

Minor: declining trend without local extinction and/or decreases less 30% at national or macro-regional level.

**Expert D**

Severe: extinction at local level or to a change of the status at national or regional level.

Moderate: evident decline observed at local (or regional) scale

Minor: probable decline not ascertained

**Expert E**

SEVERE – Negative effect bringing a strong decline at local or even global level

MODERATE – Negative effect regularly bringing density lower than expected at local scale

MINOR – Negative effect bringing minor consequences on density or breeding success

**Expert F**

SEVERE: that most likely will cause a drastic reduction of various vital parameters of ecosystems, will affect demographic parameters of local populations and will lead to conspicuous range reduction and local extinction of many species.

MODERATE: that most likely will cause significant decrease of vital parameters of some populations; which may result in loss of viability and measurable range reduction.

MINOR: that could cause some changes in the conditions of ecosystems, communities and populations; but which will not cause important reductions of population levels and will not cause conspicuous range reduction and will not lead to local extinction of species.

**Expert G**

Severe: negative impacts affecting a population and which if not controlled may cause local extinction.

Moderate: negative impacts still affecting a population which may reduce the number of individuals of a certain amount in the middle/long term (affecting density or causing a reduction in the occupied areas) but not causing extinction by itself.

Minor: still a negative impact is detectable but the amount of the reduction may be difficult to be seen or may be easily compensated by population dynamics or invasions from the neighbourhood.

**Expert H**

Severe: Local extinction, change of the status at national/ecoregional scale, severe decline (> ?? in 10 yrs)

Moderate: Decrease 10-30% in 10 yrs

Minor: declining trend without decrease < 10% in 10 YRS

**Expert I**

Severe (“very big negative effect” in the Questionnaire Instructions): determining > 30% population decline

Moderate (“big negative effect”): determining 20-30% population decline

Minor (“small negative effect”): determining low (<20%) but significant population decline

**Expert J**

Severe: determines whether high or immediate influence or on a wide area of environmental reference.

Moderate: it determines whether a direct or indirect moderate or medium scale.

Minor: If the influence is reduced or affects limited areas.

**Expert K**

Severe: local extinction risk

Moderate: extent of population decline

minor negative effects: statistical significance of declines or range contractions

**Expert L**

SEVERE: high risk of local extinction, significant population declines in a wide range

MODERATE: real risk of local extinction, significant population declines only in some areas

MINOR: low risk of local extinction, limited population declines and range contractions
