# Supplementary material for: Identifying key conservation threats to Alpine birds through expert knowledge
Source: PeerJ. 2016 Feb 29;4:e1723. doi: 10.7717/peerj.1723 (PMC4782807; doi:10.7717/peerj.1723)
Supplement: Table S1 — Tmean is the arithmetic mean of the threat score across the 19 experts who completed the survey correctly. Groups were defined according to (i) taxonomic order, which comprised Galliformes, Raptors (Accipitriformes), other non-passerines (Piciformes, Apodiformes and Cuculiformes) and Passerines; (ii) the predominant nesting habitat type used by the species in the Alps at the altitudes considered, comprising Forest (species associated with mature forest), General (generalist species occurring in a range of different habitats, often anthropogenic, and all other species which could not be classified on the basis of the former groups), Open (usually ground (or sometimes cliff)-nesting species occurring in Alpine grasslands) and Treeline (species commonly occurring in mosaic habitats around the treeline). Definitions were based on personal observations and experience, and also by the classifications used by the European Bird Census Council ( http://www.ebcc.info). SPEC is the conservation status of each species in Europe according to BirdLife’s SPEC classification (BirdLife International, 2004), where 0 indicates the species is not listed as threatened, 2 indicates a species threatened in Europe, but not globally, and most of the global breeding population is within Europe, and 3 indicates a species which is threatened in Europe but not globally, and most of the global breeding population is outside of Europe (there were no SPEC1 species). Cites is the number of published articles within a mountain environment since 1970 for a given species following a standardised search in Web of Science™ . All species were recorded at least once in point counts surveys carried out in Piedmont in 2010 to 2012 (n = 271 points) and Trentino (n = 208 points) in 2011. [file peerj-04-1723-s004.docx]

**Table S1.**  A list of all species considered in the questionnaire, and the ecological group assigned to each species. T_mean_ is the arithmetic mean of the threat score across the 19 experts who completed the survey correctly. Groups were defined according to (i) taxonomic order, which comprised Galliformes, Raptors (Accipitriformes), other non-passerines (Piciformes, Apodiformes and Cuculiformes) and Passerines; (ii) the predominant nesting habitat type used by the species in the Alps at the altitudes considered, comprising Forest (species associated with mature forest), General (generalist species occurring in a range of different habitats, often anthropogenic, and all other species which could not be classified on the basis of the former groups), Open (usually ground (or sometimes cliff)-nesting species occurring in Alpine grasslands) and Treeline (species commonly occurring in mosaic habitats around the treeline). Definitions were based on personal observations and experience, and also by the classifications used by the European Bird Census Council (http://www.ebcc.info). SPEC is the conservation status of each species in Europe according to BirdLife’s SPEC classification (BirdLife International 2004), where 0 indicates the species is not listed as threatened, 2 indicates a species threatened in Europe, but not globally, and most of the global breeding population is within Europe, and 3 indicates a species which is threatened in Europe but not globally, and most of the global breeding population is outside of Europe (there were no SPEC1 species). Cites is the number of published articles within a mountain environment since 1970 for a given species following a standardised search in Web of Science^TM^. All species were recorded at least once in point counts surveys carried out in Piedmont in 2010 to 2012 (n = 271 points) and Trentino (n = 208 points) in 2011.

| **Species** | **T_mean_** | **Taxonomic**  **group** | **Habitat**  **group** | **SPEC** | **Cites** |
| --- | --- | --- | --- | --- | --- |
| Alpine Accentor *Prunella collaris* | 0.28 | Passerines | Open | 0 | 23 |
| Alpine Chough *Pyrrhocorax graculus* | 0.31 | Passerines | Open | 0 | 29 |
| Alpine Swift *Apus melba* | 0.26 | Non-passerines | General | 0 | 31 |
| Black Grouse *Tetrao tetrix* | 1.54 | Galliformes | Treeline | 3 | 56 |
| Black Redstart *Phoenicurus ochruros* | 0.18 | Passerines | General | 0 | 12 |
| Blackbird *Turdus merula* | 0.25 | Passerines | General | 0 | 12 |
| Blackcap *Syliva atricapilla* | 0.24 | Passerines | Forest | 0 | 13 |
| Blue Tit *Cyanistes cyanus* | 0.23 | Passerines | General | 0 | 7 |
| Bonelli's Warbler *Phylloscopus bonelli* | 0.33 | Passerines | Forest | 2 | 0 |
| Bullfinch *Pyrrhula pyrrhula* | 0.28 | Passerines | Forest | 0 | 1 |
| Buzzard *Buteo buteo* | 0.65 | Raptors | General | 0 | 7 |
| Chaffinch *Fringilla coelebs* | 0.20 | Passerines | General | 0 | 9 |
| Chiffchaff *Phylloscopus collybita* | 0.25 | Passerines | Forest | 0 | 5 |
| Red-billed Chough *Pyrrhcorax pyrrhcorax* | 0.44 | Passerines | Open | 3 | 23 |
| Citril Finch *Serinus citrinella* | 0.45 | Passerines | Forest | 0 | 14 |
| Coal Tit *Periparus ater* | 0.25 | Passerines | Forest | 0 | 9 |
| Crag Martin *Ptyonoprogne rupestris* | 0.17 | Passerines | General | 0 | 0 |
| Crested Tit *Lophophanes cristatus* | 0.28 | Passerines | Forest | 2 | 2 |
| Crossbill *Loxia recuvirostra* | 0.35 | Passerines | Forest | 0 | 16 |
| Carrion/hooded Crow *Corvus corone/cornix* | 0.26 | Passerines | General | 0 | 9 |
| Cuckoo *Cuculus canorus* | 0.31 | Non-passerines | General | 0 | 3 |
| Dipper *Cinclus cinclus* | 0.43 | Passerines | General | 0 | 9 |
| Dunnock *Prunella modularis* | 0.27 | Passerines | Treeline | 0 | 5 |
| Fieldfare *Turdus pilaris* | 0.49 | Passerines | General | 0 | 2 |

Table S1. Continued.

| **Species** | **T_mean_** | **Taxonomic**  **group** | **Habitat**  **group** | **SPEC** | **Cites** |
| --- | --- | --- | --- | --- | --- |
| Firecrest *Regulus ignicappillus* | 0.28 | Passerines | Forest | 0 | 1 |
| Garden Warbler *Sylvia borin* | 0.36 | Passerines | Treeline | 0 | 4 |
| Goldcrest *Regulus regulus* | 0.29 | Passerines | Forest | 0 | 3 |
| Golden Eagle *Aquila chrysaetos* | 1.06 | Raptors | Open | 3 | 54 |
| Goldfinch *Carduelis carduelis* | 0.25 | Passerines | General | 0 | 1 |
| Greater spotted Woodpecker *Dendrocopus major* | 0.34 | Non-passerines | Forest | 0 | 1 |
| Green Woodpecker *Picus viridus* | 0.46 | Non-passerines | Forest | 2 | 0 |
| Grey Wagtail *Motacilla cinerea* | 0.33 | Passerines | General | 0 | 3 |
| House Martin *Delichon urbica* | 0.21 | Passerines | General | 3 | 0 |
| Jay *Garrulus glandarius* | 0.32 | Passerines | Forest | 0 | 6 |
| Kestrel *Falco tinnunculus* | 0.59 | Raptors | General | 3 | 3 |
| Lammergeier *Gypaetus barbatus* | 1.08 | Raptors | Open | 3 | 24 |
| Lesser Whitethroat *Sylvia curruca* | 0.37 | Passerines | Treeline | 0 | 0 |
| Linnet *Carduelis cannabina* | 0.39 | Passerines | Treeline | 2 | 1 |
| Long-tailed Tit *Aegithalos caudatus* | 0.23 | Passerines | General | 0 | 2 |
| Mistle Thrush *Turdus viscivorus* | 0.45 | Passerines | Forest | 0 | 0 |
| Nutcracker *Nucifraga caryocatactes* | 0.39 | Passerines | Forest | 0 | 12 |
| Nuthatch *Sitta europaea* | 0.35 | Passerines | Forest | 0 | 4 |
| Peregrine falcon *Falco pellegrinus* | 0.67 | Raptors | General | 0 | 16 |
| Ptarmigan *Lagopus muta* | 1.22 | Galliformes | Open | 0 | 49 |
| Quail *Coturnix coturnix* | 0.85 | Galliformes | Open | 3 | 0 |
| Raven *Corvus corax* | 0.37 | Passerines | Open | 0 | 18 |
| Red-backed Shrike *Lanius collorio* | 0.70 | Passerines | General | 3 | 8 |
| Redpoll *Carduelis flammea* | 0.29 | Passerines | Forest | 0 | 2 |
| Ring Ouzel *Turdus torquatus* | 0.65 | Passerines | Treeline | 0 | 3 |
| Robin *Erithacus rubecula* | 0.25 | Passerines | Forest | 0 | 6 |
| Rock Bunting *Emberiza cia* | 0.40 | Passerines | Treeline | 3 | 3 |
| Rock Partridge *Alectoris graeca* | 1.26 | Galliformes | Open | 2 | 18 |
| Rock Thrush *Monticola saxatilis* | 0.55 | Passerines | Treeline | 3 | 1 |
| Siskin *Carduelis spina* | 0.31 | Passerines | Forest | 0 | 0 |
| Skylark *Alauda arvensis* | 0.81 | Passerines | Open | 3 | 9 |
| Snowfinch *Montifringilla nivalis* | 0.37 | Passerines | Open | 0 | 4 |
| Song Thrush *Turdus philomelos* | 0.39 | Passerines | Forest | 0 | 3 |
| Sparrowhawk *Accipiter nisus* | 0.63 | Raptors | General | 0 | 3 |
| Swift *Apus apus* | 0.22 | Non-passerines | General | 0 | 1 |
| Tree Pipit *Anthus trivialis* | 0.58 | Passerines | Treeline | 0 | 1 |
| Treecreeper *Certhia familiaris* | 0.36 | Passerines | Forest | 0 | 2 |
| Wallcreeper *Tichodroma muraria* | 0.26 | Passerines | Open | 0 | 2 |
| Water Pipit *Anthus spinoletta* | 0.55 | Passerines | Open | 0 | 19 |
| Wheatear *Oenanthe oenanthe* | 0.57 | Passerines | Open | 3 | 8 |
| Whinchat *Saxicola rubetra* | 0.73 | Passerines | Treeline | 0 | 7 |
| White Wagtail *Motacilla alba* | 0.29 | Passerines | General | 0 | 1 |
| Willow Tit *Poecile montanus* | 0.32 | Passerines | Forest | 0 | 4 |
| Wren *Troglodytes troglodytes* | 0.23 | Passerines | Forest | 0 | 4 |
| Yellowhammer *Emberiza citrinella* | 0.57 | Passerines | Treeline | 0 | 5 |
