# Supplementary material for: Identifying key conservation threats to Alpine birds through expert knowledge
Source: PeerJ. 2016 Feb 29;4:e1723. doi: 10.7717/peerj.1723 (PMC4782807; doi:10.7717/peerj.1723)
Supplement: Table S2 — The analysis included the 39 bird species with at least one non-zero mode of threat score. [file peerj-04-1723-s005.docx]

**Table S2.** Consensus threat scores used in the NMDS analysis. The analysis included the 39 bird species with at least one non-zero mode of threat score.

| **Species** | **Abandonment** | **Climate**  **change** | **Energy** | **Forestry** | **Grazing** | **Hunting** | **Leisure** | **Urbanization** |
| --- | --- | --- | --- | --- | --- | --- | --- | --- |
| Black Grouse | 2 | 2 | 0 | 2 | 2 | 1 | 3 | 3 |
| Blackcap | 0 | 0 | 0 | 1 | 0 | 0 | 0 | 0 |
| Bonelli’s Warbler | 0 | 0 | 0 | 2 | 0 | 0 | 0 | 0 |
| Bullfinch | 0 | 0 | 0 | 1 | 0 | 0 | 0 | 0 |
| Buzzard | 1 | 0 | 1 | 1 | 0 | 1 | 0 | 1 |
| Crow | 0 | 0 | 0 | 0 | 0 | 1 | 0 | 0 |
| Dipper | 0 | 0 | 1 | 0 | 0 | 0 | 0 | 0 |
| Fieldfare | 1 | 0 | 0 | 1 | 0 | 0 | 0 | 0 |
| Garden Warbler | 0 | 0 | 0 | 1 | 0 | 0 | 0 | 0 |
| Golden Eagle | 1 | 0 | 3 | 0 | 0 | 3 | 2 | 3 |
| Goldfinch | 1 | 0 | 0 | 0 | 0 | 0 | 0 | 0 |
| Greater spotted Woodpecker | 0 | 0 | 0 | 1 | 0 | 0 | 0 | 0 |
| Green Woodpecker | 0 | 0 | 0 | 2 | 0 | 0 | 0 | 0 |
| Grey Wagtail | 0 | 0 | 1 | 0 | 0 | 0 | 0 | 0 |
| Kestrel | 1 | 0 | 1 | 0 | 0 | 0 | 0 | 0 |
| Lammergeier | 3 | 0 | 1 | 0 | 0 | 3 | 3 | 3 |
| Lesser Whitethroat | 0 | 0 | 0 | 1 | 0 | 0 | 0 | 0 |
| Linnet | 2 | 0 | 0 | 0 | 1 | 0 | 0 | 0 |
| Mistle Thrush | 1 | 0 | 0 | 1 | 0 | 0 | 0 | 0 |
| Peregrine falcon | 0 | 0 | 1 | 0 | 0 | 2 | 2 | 1 |
| Ptarmigan | 0 | 3 | 0 | 0 | 2 | 3 | 3 | 3 |
| Quail | 3 | 2 | 0 | 0 | 1 | 1 | 0 | 1 |
| Red-backed Shrike | 3 | 0 | 0 | 0 | 1 | 0 | 0 | 2 |
| Ring Ouzel | 1 | 1 | 0 | 0 | 0 | 0 | 0 | 0 |
| Robin | 0 | 0 | 0 | 1 | 0 | 0 | 0 | 0 |
| Rock Bunting | 0 | 0 | 0 | 0 | 1 | 0 | 0 | 0 |
| Rock Partridge | 2 | 2 | 0 | 0 | 2 | 3 | 3 | 3 |
| Rock Thrush | 2 | 0 | 0 | 0 | 1 | 0 | 0 | 0 |
| Skylark | 3 | 0 | 0 | 0 | 1 | 0 | 0 | 0 |
| Snowfinch | 0 | 3 | 0 | 0 | 0 | 0 | 0 | 0 |
| Sparrowhawk | 0 | 0 | 0 | 1 | 0 | 1 | 0 | 0 |

**Table S2.** Continued.

| **Species** | **Abandonment** | **Climate**  **change** | **Energy** | **Forestry** | **Grazing** | **Hunting** | **Leisure** | **Urbanization** |
| --- | --- | --- | --- | --- | --- | --- | --- | --- |
| Tree Pipit | 2 | 0 | 0 | 1 | 0 | 0 | 0 | 0 |
| Treecreeper | 0 | 0 | 0 | 1 | 0 | 0 | 0 | 0 |
| Water Pipit | 1 | 0 | 0 | 0 | 1 | 0 | 0 | 0 |
| Wheatear | 1 | 0 | 0 | 0 | 1 | 0 | 1 | 0 |
| Whinchat | 3 | 0 | 0 | 0 | 3 | 0 | 1 | 0 |
| White Wagtail | 1 | 0 | 0 | 0 | 0 | 0 | 0 | 0 |
| Wren | 0 | 0 | 0 | 1 | 0 | 0 | 0 | 0 |
| Yellowhammer | 3 | 0 | 0 | 0 | 1 | 0 | 0 | 2 |

Threats are defined in Table 1. A consensus score across respondents for each species/threat combination was obtained by calculating the mode of the scores across different respondents. Mining and fire were not included due to low concordance across experts (Table 2).
